# Seroprevalence of *Toxoplasma gondii* in Wild European and American Mink (*Mustela lutreola* and *Neogale vison*) from Spain

**DOI:** 10.3390/pathogens14050427

**Published:** 2025-04-28

**Authors:** María Eugenia Lebrero, José Villora, María Asunción Gómez, Madis Podra, María del Carmen Aranda, Sergio Villanueva-Saz, Antonio Fernández, Patricia Lizarraga, Pablo Quilez, Álex Gómez, Diana Marteles

**Affiliations:** 1 Department of Animal Pathology, Veterinary Faculty, University of Zaragoza, Calle Miguel Servet, 177, 50013 Zaragoza, Spain; wlebrero@unizar.es (M.E.L.); josevilloragon@gmail.com (J.V.); afmedica@unizar.es (A.F.); pquilezloz1109@gmail.com (P.Q.); a.gomez@unizar.es (Á.G.); 2 Clinical Immunology Laboratory, Veterinary Faculty, University of Zaragoza, Calle Miguel Servet, 177, 50013 Zaragoza, Spain; 3 Tragsatec, Tragsatec, Division of Environmental Services, Julian Camarillo 6A-4A Planta, Sector B, 28037 Madrid, Spain; asun_emink@yahoo.es (M.A.G.); madis.podra@yahoo.es (M.P.); maranda4@tragsa.es (M.d.C.A.); 4 Fundación para la Investigación en Etología y Biodiversidad, Casarrubios del Monte, 45950 Toledo, Spain; 5 Instituto Agroalimentario de Aragón-IA2 (Universidad de Zaragoza-CITA), Calle Miguel Servet, 177, 50013 Zaragoza, Spain; 6 Centro de Recuperación de Fauna de Martioda, Martioda Entitatea, 3, 01191 Martioda, Spain; lizarmen79@gmail.com

**Keywords:** *Toxoplasma gondii*, European mink, American mink, indirect immunofluorescence

## Abstract

Toxoplasmosis, caused by the intracellular parasite *Toxoplasma gondii*, affects a wide range of warm-blooded animals, including humans. Domestic and wild felines serve as definitive hosts, excreting oocysts that contaminate the environment. Intermediate hosts, such as the endangered European mink (*Mustela lutreola*) and the non-native American mink (*Neogale vison*), can become infected primarily through the ingestion of tissue cysts present in prey, while the ingestion of oocysts from contaminated soil or water plays a secondary role. This study analyzed the seroprevalence of *T. gondii* in 179 mink specimens (137 American mink and 42 European mink) collected in northern Spain from 2014 to 2020. Using an optimized indirect immunofluorescence assay, antibodies against *T. gondii* were detected in 47 samples (37 American mink and 10 European mink). Seroprevalence was higher in the Ebro basin than in the Cantabrian region, although the difference was not statistically significant. No significant associations were observed between seropositivity and species, sex, or habitat. These findings suggest environmental contamination by *T. gondii* oocysts in northern Spain and underscore the potential value of invasive American mink as sentinel species for monitoring public health risks associated with this parasite. The study also highlights the importance of wildlife surveillance in assessing environmental contamination and understanding transmission dynamics of infectious diseases in ecosystems.

## 1. Introduction

Toxoplasmosis is a globally distributed disease that can affect any warm-blooded animal species, including humans [1,2,3,4,5]. It is caused by the obligate intracellular parasite *Toxoplasma gondii* [1]. Domestic and wild carnivores are the only definitive host; all other animal species can serve as intermediate hosts. In the case of domestic and wildfelines, they can play a dual role as final and intermediate hosts [6,7].

Carnivores, such as minks, become infected primarily through the tissues of other intermediate hosts. The ingestion of oocysts excreted exclusively in feline feces, which contaminate food, soil, or water, plays a secondary role [8,9,10]. These oocysts can remain viable in the environment for months, depending on temperature and humidity [6,11]. Transmission can also occur via the ingestion of tissue cysts found in the organs and tissues of infected animals; these cysts contain infectious forms of the parasite, such as bradyzoites. Carnivores typically exhibit higher seroprevalence, due to consuming infected meat, and transplacental transmission has likewise been documented [3,12,13,14].

In immunocompetent hosts, toxoplasmosis generally remains subclinical [3,8]. However, it can affect the respiratory, nervous, and ocular systems [15]. In females, toxoplasmosis may lead to reproductive disorders, including abortions, and can cause lesions or mortality in neonates [9]. Toxoplasmosis is particularly significant in wild animals kept in captivity. For instance, in captive mustelids, this disease has been associated with considerable economic losses on farms [2,4,16,17,18], though such cases are often sporadic and secondary to immunosuppressive conditions such as distemper [2,14,17,19].

The detection of antibodies in herbivore species is a valuable indicator of environmental contamination by oocysts [20,21,22]. This study focuses on two species of high conservation importance: the endangered European mink and the invasive American mink. The European mink population has declined significantly, whereas efforts to control the American mink have positively influenced the conservation of the European mink. Similar to other endangered species, such as the Iberian lynx (*Lynx pardinus*), the European mink faces multiple threats, including habitat destruction and fragmentation, prey decline, roadkill, and potentially various infectious and parasitic diseases [23,24].

Among parasitic pathogens, *T. gondii* remains understudied in endangered species within the Iberian Peninsula. Recently, an epidemiological investigation in central Spain collected 69 samples from Iberian lynxes and 20 from wildcats (*Felis silvestris*). Seroprevalence was found to be 85% in wildcats and 45% in lynxes, as determined by the Indirect Fluorescent Antibody Test (IFAT) [24]. The analysis of seroprevalence in wild felines does not indicate a risk of oocyst shedding and environmental contamination; rather, this seropositivity may result from infection as an intermediate host.

From an animal conservation perspective, it is essential to understand how invasive species influence disease control, particularly concerning zoonotic diseases. In this regard, wild raccoons (*Procyon lotor*) and raccoon dogs (*Nyctereutes procyonoides*) in Poland have shown seropositivity rates of 48.73% and 28.09%, respectively, indicating a high level of parasite circulation in the environment [25].

In our study, we assessed toxoplasmosis in both threatened and invasive alien species in the wild. In Spain, the critically endangered European mink and the alien American mink coexisted: the European mink survived within a limited area in the northern part of the country, whereas the American mink, a primary threat to the native species, occupied a broad region across the northern and central Iberian Peninsula [26]. Originally introduced from North America for fur farming, American mink have either escaped or been intentionally released, leading to their broad distribution. As an invasive species in Europe, the American mink poses a significant threat to the native European mink through both direct competition and predation.

The aim of this study was to analyze the seroprevalence of *Toxoplasma gondii* in European and American minks in northern Spain from 2014 to 2020 and to evaluate the parasite’s potential impact on the conservation of the protected European mink, as well as on the alien American mink.

## 2. Materials and Methods

### 2.1. Animals

**Figure 1:**
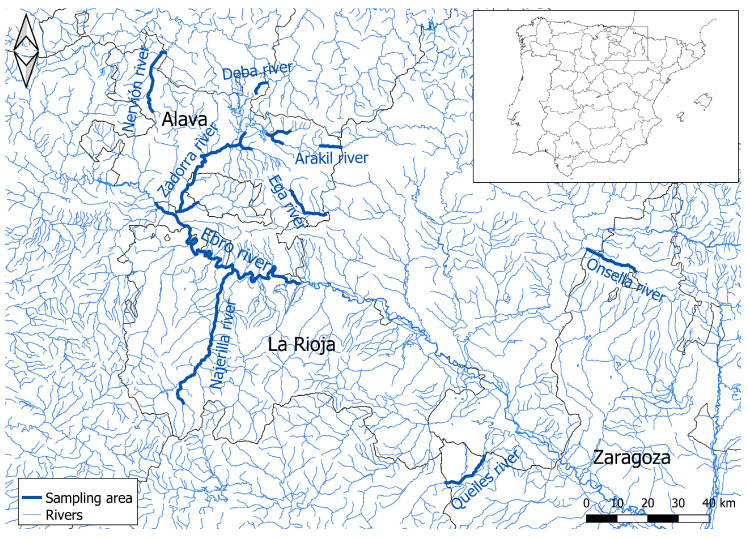
Location of sampling area from the Cantabrian and Ebro basins, northern Spain, 2014–2020. The main map shows the region of Castilla y León, La Rioja, País Vasco, and Aragón.

During this period, 179 mink specimens were examined, including 137 American mink (*Neogale vison*) and 42 European mink (*Mustela lutreola*), all collected in northern Spain. Of these, 157 were captured from rivers in the Ebro basin (112 American mink and 42 European mink), while an additional 25 American mink were collected from rivers in northern Spain (Figure 1).

Samples were obtained during the implementation of European mink conservation programs conducted by regional governments and the Ministry for the Ecological Transition and the Demographic Challenge in Spain, with support from the European Commission LIFE program (LIFE LUTREOLA SPAIN). American mink samples were collected during population eradication operations within the European mink distribution area, whereas European mink samples were gathered during field surveys for population monitoring. The care and use of all animals adhered to the Spanish Animal Protection Policy (RD 53/2013) and complied with European Union Directive 2010/63 on the protection of animals used for experimental and other scientific purposes.

Both species were captured using single-entry wire cage traps (15 × 15 × 60 cm). European mink were anesthetized intramuscularly with ketamine (5 mg/kg; Imalgene 1000, Merial, Lyon, France) and medetomidine (0.10 mg/kg; Domtor, Orion Corporation, Espoo, Finland), and blood samples were collected via jugular puncture. After recovering from anesthesia, these animals were released at their capture sites. Similarly, American minks were anesthetized, and blood samples were collected by jugular vein puncture or cardiac puncture. These animals were subsequently euthanized in accordance with legal animal welfare regulations, as the species is classified as an exotic invasive species.

### 2.2. Serological Technique

**Figure 2:**
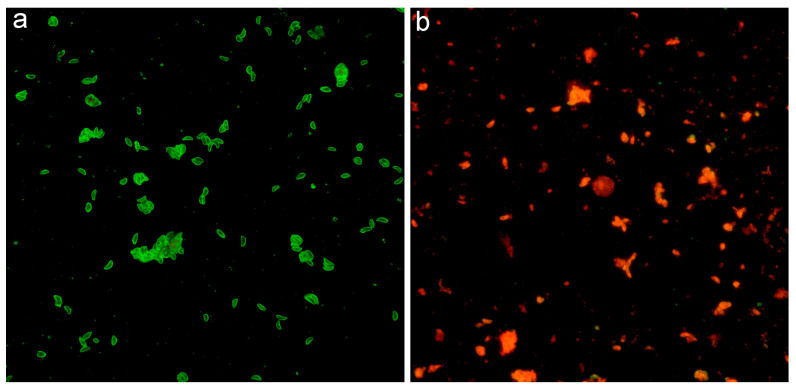
Immunofluorescence pattern detected by IFAT (×40): (**a**) Positive fluorescence pattern. The *Toxoplasma* tachyzoites show a bright, sharp, clear, yellow-green fluorescence; (**b**) Negative fluorescence pattern. There is no clear fluorescence but a weak red-greyish color of the *Toxoplasma* tachyzoites.

The IFAT was performed following the standard procedures of the World Organization for Animal Health [27]. Tachyzoite forms were used as whole-parasite antigens (Complutense University of Madrid, Spain), fixed on multi-spot slides (Thermo Fisher Scientific, Waltham, MA, USA). To detect *T. gondii* antibodies, sera were diluted 1:32 in phosphate-buffered saline (PBS). Briefly, a twofold serial dilution of each serum was applied to each well. The slides were incubated for 30 min at 37 °C in a humid chamber, washed twice with PBS for 5 min, and rinsed with distilled water. After washing, 20 µL of FITC-Protein A conjugate (Sigma-Aldrich, Saint Louis, MO, USA) diluted at a 1:32 ratio in 0.2% Evans blue dye was added to each well. Protein A was used as a conjugated reagent due to its ability to bind the fragment crystallizable region of immunoglobulin G from various animal species. This reagent has been employed in serological studies for detecting antibodies against multiple pathogens, including *Leishmania infantum*, *Dirofilaria immitis*, and SARS-CoV-2, in species such as dogs [28], cats [29], and mustelids such as ferrets, European mink, and American mink [30,31,32]. Slides were incubated at 37 °C for an additional 30 min in complete darkness and then washed again as described. Following the second washing step, mounting medium was applied, and slides were examined under a fluorescence microscope (Leica DM750 RH; Leica Microsystems, Wetzlar, Germany) at 400× magnification. Positive controls displayed tachyzoites with bright, sharp, yellow-green fluorescence on their membranes (Figure 2a), while negative controls appeared grayish-dark red, lacking clear fluorescence (Figure 2b). Each IFAT sample was analyzed independently by two trained researchers, with a third investigator resolving any discrepancies. The cut-off for the IFAT was established at a 1:32 dilution by analyzing 90 serum samples from European mink classified as healthy, based on annual healthcare assessments conducted between 2021 and 2023. These routine evaluations included comprehensive physical examinations, blood cell counts, and biochemical profiles. The samples collected from European mink in the National Captive-Breeding Program at the Foundation for Research in Ethology and Biodiversity (FIEB) breeding center in Casarrubios del Monte (Toledo, Spain) were not included in this study but were used solely to define the cut-off. Because positive reference serum samples from mink were unavailable, the IFAT protocol was validated through parallel testing with canine, feline, and ferret sera. Positive controls were obtained from seropositive samples identified for diagnostic purposes, while negative controls consisted of samples from healthy, non-infected individuals of these species.

### 2.3. Statistical Analysis

Data were analyzed using SPSS version 28 (SPSS Inc., Chicago, IL, USA). Descriptive analyses were performed for variables such as sex, geographic origin (Ebro or Cantabrian basin), and species. Fisher’s exact test, with 95% confidence intervals (CI), was used to compare proportions. The seroprevalence of *T. gondii* was calculated along with 95% CIs, and statistical significance was set at *p* < 0.05.

## 3. Results

Of the 179 serum samples analyzed, anti-*Toxoplasma* antibodies were detected in 47, while 132 tested negative. Among the positive samples, 37 were from *N. vison* (n = 137), and the remaining 10 were from *M. lutreola* (n = 42). Regarding sex-related seropositivity in *N. vison* mink, 17 out of 70 females and 20 out of 67 males tested seropositive. For *M. lutreola*, 6 out of 23 females and 4 out of 19 males were seropositive. No statistically significant differences were observed between species.

When examining American mink samples by geographic origin, a higher number of seropositive animals were found in the Ebro basin (34/112) compared to the Cantabrian region (3/100). For European mink, a total of 10 seropositive samples were identified, all located in the Ebro basin. No statistically significant differences were detected between geographic origin and seropositivity in American mink.

## 4. Discussion

Concerning American mink, studies on wild carnivorous mammals in Poland and the United Kingdom corroborate our findings, reporting seroprevalence rates of 25% and 29.2%, respectively, as determined by PCR [5,33]. Ribas et al. (2018) noted a seropositivity rate of 78.8% in northern Spain between 2010 and 2015, using a modified agglutination test [34]. These results are also consistent with data from feline species in Spain, where seroprevalence rates of 84.7% by the modified agglutination test (MAT) was documented in 2009 and 2018, respectively [35].

Of the 179 serum samples analyzed in this study, anti-*Toxoplasma* antibodies were detected in 47, while 132 tested negative. Of the 47 positive samples, 37 were from *Neovison vison* (n = 137) and 10 were from *Mustela lutreola* (n = 42). No statistically significant differences were observed between species. When examining American mink by geographic origin, a higher number of seropositive animals were found in the Ebro basin (34/112) compared to the Cantabrian region (3/100). For European mink, 10 positive samples were identified, all of which were located in the Ebro basin. No statistically significant differences (*p* > 0.05) were detected between geographic origin and seropositivity in American mink.

The seroprevalence detected in American mink in Chile in 2007 and 2013 was 70% and 59%, respectively, and 77% in the United States, as determined using agglutination-based diagnostic techniques [11,36,37]. More recently, Heddergott et al. reported a seroprevalence of 45.36% in wild mink from Poland and Germany between 2019 and 2022, based on enzyme-linked immunosorbent assay (ELISA) [38]. Differences in seroprevalence across these studies, compared to the present one, may be explained by variations in climatic conditions, habitat, and mammal communities.

Although a higher percentage of positive samples was observed among American mink from the Ebro basin (30.6%) than from the Cantabrian region (12%), this difference was not statistically significant (*p* = 0.091). Similarly, a study on American mink in northern Spain found no significant differences in seroprevalence between mink from different river basins, including the Ebro and Duero [34]. These studies reported significantly higher seroprevalence rates in areas with medium to high domestic cat presence, including urban and peri-urban regions characterized by higher cat densities [36]. This factor was not considered in the present study, but may account for the discrepancies with earlier findings [34] or among different rivers.

In the current study, of the 47 positive samples, 23 were from females and 24 from males, with no statistically significant differences (*p* > 0.05). Similarly, Ribas et al. found no association between sex and seropositivity in northern Spain, nor did studies in southern Chile, Germany, and Poland [34,36,38]. By contrast, another study reported higher seroprevalence in wild male mink than in females [39]. This variation could be attributed to differences in diet, as males primarily hunt medium-sized mammals, whereas females mainly consume fish and crustaceans [40]. Since fish do not develop tissue cysts, they likely play a negligible role in the epidemiology of *T. gondii* [41]. Moreover, males tend to make longer movements and have larger territories, potentially increasing both direct and indirect contacts with other potential hosts.

In animals, the most commonly employed serological techniques for the detection of *T. gondii* antibodies include IFAT, ELISA, and various agglutination methods [42]. However, comparative data on the diagnostic performance of these assays in animal hosts remain limited. According to a recent systematic review and meta-analysis [43], indirect ELISA, particularly when using native or chimeric recombinant antigens, demonstrated high diagnostic accuracy, with pooled sensitivity and specificity values reaching 98.7 percent and 99.2 percent, respectively. These results indicate that ELISA is among the most reliable serological tools available for diagnosing *T. gondii* infection.

In our study, we employed IFAT as the diagnostic method, which is widely accepted in veterinary seroepidemiology due to its high specificity. Although this technique was not quantitatively analyzed in the aforementioned meta-analysis, it remains a well-established method in the field. Its application, however, requires fluorescence microscopy and experienced personnel, which may affect reproducibility across laboratories. According to the World Organisation for Animal Health (WOAH), IFAT is considered an appropriate diagnostic method, though further validation may be necessary [27]. In our study, we addressed this need by including a *T. gondii*-free mink population as a negative control in order to establish an appropriate cut-off value.

Several studies have highlighted the variability in diagnostic performance across serological techniques. For instance, it has been reported that the MAT test may exhibit higher sensitivity than ELISA in specific cases [44]. In the present review, ELISA and IFAT were the most commonly applied techniques to assess *T. gondii* seroprevalence. Despite potential limitations in sensitivity and specificity, serological methods remain standard tools for the qualitative detection of anti-*T. gondii* antibodies [45].

Furthermore, a recent study comparing three serological techniques in naturally infected wolverines (*Gulo gulo*) found that ELISA and IFAT exhibited relatively higher sensitivity and specificity compared to MAT. The same study also noted that ELISA and IFAT were less labor-intensive and time-consuming, further supporting their practical utility in epidemiological investigations [46]. Finally, immunochromatographic tests have been developed; however, these types of rapid tests can be used in cats, but they are not valid to other animal species due their specific anti-cat IgG [47], whilst the quantitative serological technique is possible to use different specific FITC-conjugates for IFAT and enzyme-conjugates for ELISA for each animal species evaluated [48].

The MAT is the most commonly used diagnostic method in seroprevalence studies of mustelids [11,37,38,49,50]. However, a 2013 study conducted in northeastern China that compared *T. gondii* seroprevalence in American mink farms using multiple laboratory techniques, including MAT, Western blot, and ELISA, concluded that MAT often yields false negatives in mink, possibly due to its limited species specificity [4]. In the present study, the IFAT test was selected for minks because it is the preferred technique for determining prevalence in cats [8] and in other wild felines [24].

Molecular diagnostic techniques such as polymerase chain reaction (PCR), nested PCR, real-time PCR, loop-mediated isothermal amplification (LAMP), multiplex PCR, PCR-restriction fragment length polymorphism (PCR-RFLP), microsatellite DNA analysis, and multilocus DNA sequence typing of introns are available for the detection of *T. gondii* infection. These methods provide highly sensitive detection of protozoan infections in animals, allowing for early diagnosis, even before the onset of antibody production. Furthermore, PCR-based detection of the parasite in peripheral blood is particularly valuable. It enables confirmation of parasitemia in infected hosts, especially during systemic dissemination of the protozoan or recrudescence of encysted bradyzoites, which may coincide with the emergence of clinical signs.

Several molecular techniques have been employed to investigate the genetic composition of *T. gondii* strains [51], including PCR-RFLP [52], microsatellite DNA analysis [53], and multilocus DNA sequence typing of introns [54]. An important advantage of these molecular methods is their ability to enable both species identification and genotyping of *T. gondii*. This is essential for assessing intraspecific genetic diversity and exploring the genetic background of different strains. Notably, *T. gondii* exhibits a clonal population structure [55]. The most frequently isolated strains worldwide, particularly in Europe and North America, belong to three main clonal lineages, designated as types I, II, and III. Additional clonal lineages, referred to as atypical or unique genotypes, have also been identified. These are often associated with specific geographic regions and are distinct from the canonical lineages. The significance of *T. gondii* infection varies depending on the host species and parasite genotype. Type I strains are typically uniformly lethal in mice, while type II and III strains are considerably less virulent. In humans, clinical manifestations of infection range from asymptomatic to severe acute toxoplasmosis, with type II being the most commonly identified genotype [56,57,58].

The presence of anti-*Toxoplasma* antibodies in wild minks may reflect exposure through infection as an intermediate host. Today, the alien American mink is widespread in Spain and many other European countries, and it continues to be used in fur farming [26,59]. Consequently, feral minks and farmed populations of American mink may, therefore, function as sentinel species for this disease.

By contrast, the critically endangered European mink is unlikely to substantially impact public health. Instead, toxoplasmosis and other diseases may pose additional threats to its already vulnerable populations in the wild, underscoring the need for vigilant health monitoring and disease management, particularly in captive breeding programs aimed at species conservation.

## Data Availability

The datasets generated during and/or analyzed during the current study are available from the corresponding author on reasonable request.

## Abbreviations

The following abbreviations are used in this manuscript:

IFAT	Indirect fluorescent antibody test
FIEB	Research in ethology and biodiversity
MAT	Modified agglutination test
IFI	Indirect immunofluorescent test
ELISA	Enzyme-linked immunosorbent assay
WOAH	World Organization for Animal Health
